# Mammary candidiasis: A medical condition without scientific evidence?

**DOI:** 10.1371/journal.pone.0181071

**Published:** 2017-07-13

**Authors:** Esther Jiménez, Rebeca Arroyo, Nivia Cárdenas, María Marín, Pilar Serrano, Leonides Fernández, Juan M. Rodríguez

**Affiliations:** 1 Dpt. Nutrition, Food Science and Food Technology, Complutense University of Madrid, Madrid, Spain; 2 Unidadde Endocrinología y Nutrición, Hospital Virgen del Rocío, Seville, Spain; Wageningen University, NETHERLANDS

## Abstract

Many physicians, midwives and lactation consultants still believe that yeasts (particularly *Candida* spp.) play an important role as an agent of nipple and breast pain despite the absolute absence of scientific proofs to establish such association. In this context, the objective of this study was to investigate the microorganisms involved in sore nipples and/or painful “shooting” breastfeeding by using a variety of microscopy techniques, as well as culture-dependent and–independent identification methods. Initially, 60 women (30 diagnosed as suffering “mammary candidiasis” and 30 with no painful breastfeeding) were recruited to elucidate the role of their pumps on the milk microbial profiles. After realizing the bias introduced by using such devices, manual expression was selected as the collection method for the microbiological analysis of milk samples provided by 529 women with symptoms compatible with “mammary candidiasis”. Nipple swabs and nipple biopsy samples were also collected from the participating women. Results showed that the role played by yeasts in breast and nipple pain is, if any, marginal. In contrast, our results strongly support that coagulase-negative staphylococci and streptococci (mainly from the mitis and salivarius groups) are the agents responsible for such cases. As a consequence, and following the recommendations of the US Library of Medicine for the nomenclature of infectious diseases, the term “mammary candidiasis” or “nipple thrush” should be avoided when referring to such condition and replaced by “subacute mastitis”.

## Introduction

Lactation is probably the only bodily function for which modern medicine has almost no training, protocol or scientifically-acquired knowledge. When women suffer painful breastfeeding, they usually have to face the dilemma of continuing despite the pain or giving it up, preventing mothers and infants from getting the well-recognized health benefits associated to breastfeeding [1–3]. In practice, it is very unusual that they are offered the option of clinical tests (including milk analysis and antibiogram) in order to know the pain etiopathogenesis or to guide the therapeutic approach.

This situation has contributed to the spread of non-scientific beliefs to explain the origin of painful breastfeeding and to the maintenance of traditional, and often useless, empirical treatments. One of such beliefs is that yeasts (mainly *Candida albicans*) play an important role in nipple and breast pain. In the absence of fever or flu-like symptoms, the onset of sore burning, painful nipples or radiating or “shooting” pain into the axilla in breastfeeding women has traditionally been diagnosed and treated as “ductal or mammary candidiasis” by many physicians, midwives and lactation consultants [4–9]. Such diagnosis is made in virtually all cases by visual assessment of the breast, without supporting laboratory findings and ignoring that the evidence of a potential association *Candida*-painful breastfeeding is largely anecdotal [10,11]. Recently, studies applying milk microbial analysis (including some examining for the presence of yeasts) have shown that coagulase-negative staphylococci (CNS), streptococci (mitis and salivarius groups) and corynebacteria may be actually the agents responsible for such symptoms [12–15]. However, studies directed to specifically and systematically investigate the presence or absence of *Candida* cells and/or DNA in a large collection of milk samples from women with these symptoms are very scarce.

In this context, the objective of this work was to elucidate the actual etiology of 529 cases of sore nipples and/or painful breastfeeding, initially diagnosed as “ductal or mammary candidiasis”, by using a great variety of microscopy techniques, as well as culture-dependent and–independent identification methods applied to milk, nipple swabs and nipple biopsy samples.

## Materials and methods

### Participating women and collection of the samples

**Fig 1:**
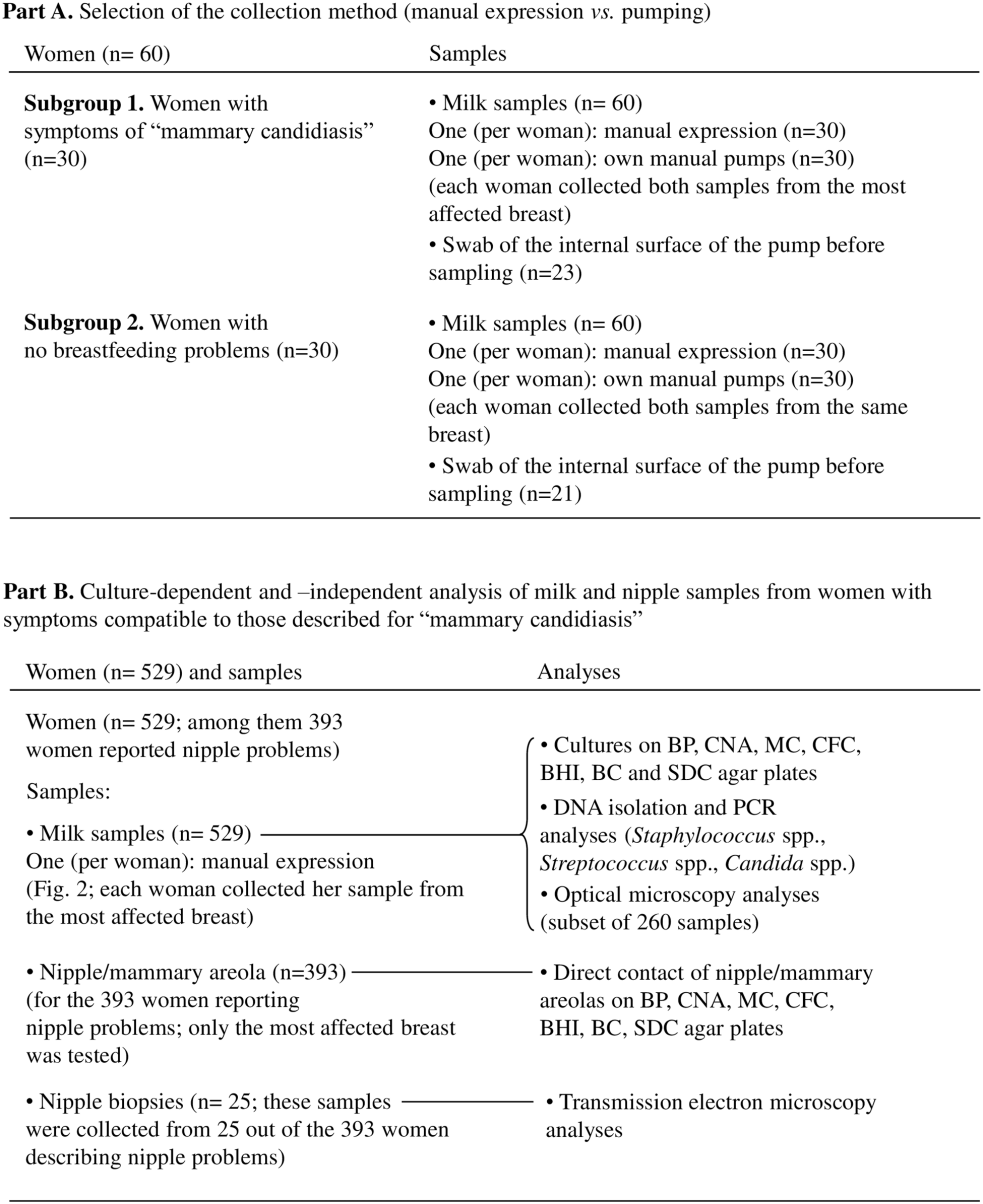
Schematic representation of the two parts of the study, including the number of recruited women and the type and number of the biological samples that were analysed.

Globally, the number of women and the type and number of the samples that were analyzed in this study are shown in Fig 1. Initially, 60 women were recruited in order to elucidate the contribution of milk pumps on the milk microbial load and profile. Among them, 30 women displayed breast/nipple symptoms traditionally associated to “mammary candidiasis” and 30 women had no breastfeeding problems. These women collected one sample obtained by manual expression following the protocol described by Arroyo et al. [16], and a second one by using their own manual milk pump. The internal surfaces of the pumps were also sampled (Fig 1) using a sterile swab, in order to ascertain their role in bacterial milk load. Sample collection was supervised by midwives, gynecologists, pediatricians or nurses.

After realizing the strong effect of pumps on the milk microbial pattern, manual expression was selected as the only collection method for further microbiological analyses. Thus, 529 women with symptoms compatible to those related to “mammary candidiasis” were subsequently recruited from July 2011 to July 2015. All of them reported painful breastfeeding with radiating or “shooting” pain into the axilla or the back and 393of these 529 women (74%) also reported sore, burning, or painful nipples. Women with symptoms of acute mastitis (including breast redness, fever and other flu-like symptoms), Raynaud’s disease or breast abscess were excluded from this study. Neither antibiotics nor probiotics were administered to the women prior to the collection of samples. Each woman provided a milk sample from her most severely affected breast (529 samples), which were subsequently submitted to culture, PCR and optical microscopy analyses (Fig 1). A subset of women reporting painful nipples(n = 393) were asked to have a direct contact of the nipple and mammary areola of their most affected breast by pressing such structures on different agar media (see below). In addition, 25 women of the same 393 subset provided a nipple biopsy sample, and such samples were processed as explained below.

### Ethics statement

All volunteers gave written informed consent to the protocol (reference 10/017E), which had been previously approved by the Ethical Committee of Clinical Research of Hospital Clínico San Carlos Madrid (Spain).

### Cultures and identification of isolates

Immediately after collection, milk samples and swabs taken from the internal surfaces of the pumps were plated onto Baird Parker (BioMérieux, Marcy l’Etoile, France; isolation of staphylococci), Columbia Nadilixic Acid (CNA, BioMérieux; isolation of staphylococci, streptococci, enterococci, corynebacteria and related Gram-positive bacteria; this medium also allows yeasts’ growth), MacConkey (MC, BioMérieux; isolation of enterobacteria), Pseudomonas Agar Base (CFC formulation, Oxoid, Basingstoke, UK; isolation of *Pseudomonas*, *Stenotrophomonas* and related Gram-negative bacteria), Brain Heart Infusion (Oxoid; general medium for bacterial and fungal growth), Brilliance Candida (BC, Oxoid; isolation and preliminary identification of *Candida* species), and Sabouraud Dextrose Chloramphenicol (SDC, BioMérieux; isolation of yeasts) agar plates in order to identify and quantify the bacteria or the yeasts present in the samples. In addition, women reporting painful nipples(n = 393) were asked to have a direct contact of the nipple and mammary areola of the most affected breast on CNA, BC and SDC agar plates. All the media were incubated following the manufacturers’ recommendations.

The isolates (at least 5 isolates of each colony morphology per medium and per sample) were identified by Matrix Assisted Laser Desorption Ionization-Time of Flight (MALDI-TOF) mass spectrometry using a Vitek-MS^™^ instrument (BioMérieux) in the facilities of Probisearch (Tres Cantos, Madrid, Spain). Briefly, a portion of a microbialcolony (~1 μL) was directly spotted onto a MALDI sample plate. Then, it was overlaid with 1 μL of a saturated solution of α-cyano-4-hydroxycinnamic acid in acetonitrile (28%, w/v), and allowed to dry at room temperature. For each isolate, a mean spectrum was constructed with at least 50 *m/z* spectra profiles and used for the identification by comparison with the spectra contained in the Myla database (BioMérieux). Identification was defined as a 99–100% match to the species-specific *m*/*z* values in the database. Those isolates belonging to the species *Streptococcus mitis* or *Streptococcus oralis* were classified as *Streptococcus mitis/oralis* since MALDI-TOF analysis were not able to discriminate between them.

### DNA isolation from human milk

Samples (2 mL) were centrifuged at 11,000×*g* for 5 min at 4°C; the pellet was washed with TE buffer (10 mM Tris; 1mM EDTA; pH 8), and suspended in 0.5 mL of extraction buffer (200 mM Tris–HCl pH 7.5, 0.5% (w/v) SDS, 25 mM EDTA, 250 mM NaCl, 20 mg/mL lysozyme, 5 μg/mL lysostaphin) and 0.3 mL of 3 M sodium acetate. Then, mechanical lysis was performed three times by bead-beating with 0.1 mm diameter zirconia/silica beads (Sigma-Aldrich Quimica SL, Madrid, Spain) using a FastPrep disruptor (QBioGene, Irvine, CA, USA) at a speed setting of 6.0 m/s for 30 s. The lysate solution was treated with 0.1 mg/mL of proteinase K (Sigma), and incubated for 30 min at 37°C. Following incubation, 0.1 mL of 1.5 M NaCl was added to the lysate and mixed. After incubation for 5 min at room temperature, the mixture was centrifuged at 16,000 ×*g* to pellet the insoluble cell debris. The supernatant was transferred into a new tube and extracted twice with an equal volume of phenol/chloroform/isoamyl-alcohol (25:24:1) (Sigma). The DNA was precipitated by adding 0.6 volumes of isopropanol (Sigma) and incubating at -20°C for 1 h. The DNA pellet was washed with 70% ethanol, allowed to air-dry, and finally suspended in TE buffer. The DNA yield was measured using a NanoDropH ND-1000 UV spectrophotometer (Nano-Drop Technologies, Wilmington, DE). A concentration of ≥500 ng of DNA was obtained from the different samples. Parallel, a mechanical disruption method previously used for PCR detection of *Candida* spp. was also used following the authors’ instructions [17].

The PCR technique was used to detect *Staphylococcus* and *Streptococcus* DNA present in the samples using primers pairs and PCR conditions described previously [18]. In addition, PCR amplification of *Candida* spp. sequences was performed as described by Shin *et al*. [17].

### Optical microscopy analysis of milk samples

The May-Grünwald-Giemsa stain [19] was used for the optical microscopy study of cells in the fresh human milk samples. The samples (10μL of milk or a nipple swab) were spread on a slide forming a square (~1 cm^2^). Once dry, the spread sample was fixed with methanol and air-dried. The slide was sequentially submerged in a May-Grünwald solution at 50% in PBS for 2 min, in a pure May-Grünwald solution for 3 min, and in a Giemsa solution at 10% in PBS for 10 min. Finally, the slide was rinsed with water to eliminate the remaining staining solution and left to dry before microscope observation. The different types of cells were differentiated at 100×.

### Transmission electron microscopy (TEM) analysis of nipple biopsies

Nipple biopsies (from 25 cases of sore/painful nipples) were fixed in 4% (v/v) paraformaldehyde and 2.5% (v/v) glutaraldehyde (EMS, Hatfield, Pennsylvania, USA) in PBS pH 7.2 for 4 h at 4°C. The samples were washed with cold sterile PBS every 20 minutes, at least 4 times. Biopsies were then post-fixed with a 1% (w/v) EM grade osmium tetroxide solution for 90 min at room temperature. This was followed by washing with sterile deionized water 4 times every 15 minutes and a complete dehydration of the specimens in a series of increasing acetone concentrations (30, 50, 70, 80, 90, 95 and 100%). Then, the samples were infiltrated and embedded gradually in epoxy resin. Ultrathin sections were obtained in an Ultracut E microtome (Reichert Jung, Buffalo, New York, USA) and stained with 2% (w/v) uranyl acetate followed by Reynold´s lead citrate. Electron micrographs were obtained with a JEOL 1010 electron microscope at 100 kV and equipped with a CCD megaview camera at the Microscopy National Center (Complutense University of Madrid, Spain).

### Statistical analysis

Microbiological data, recorded as colony-forming unit (CFU)/mL, were transformed to logarithmic values before statistical analysis. Microbial counts were tested for normality by Shapiro-Wilk tests. Levene’s tests were used to assess the homogeneity of variances. The reported values of microbial counts are the mean values and 95% confidence interval (CI) of the mean or, when they were not normally distributed, as the median and the interquartile range (IQR). Frequencies of detection of each microbial species or group were compared using the Fisher exact test. Microbial countsin milk samples obtained from women with and without symptoms of presumptive mammary candidiasis after milk extraction by either manual expression or pumping were analysed by two-way ANOVA tests to determine the effect of pain (present or absent) and type of milk expression (manual and use of pump) and their interaction. One-way ANOVA tests were used to test for differences amongst the means of microbial counts in milk samples obtained after manual or pump expression from both groups of participants (women with and without painful breastfeeding). Similarly, mean microbial counts in milk samples provided by women with and without painful breastfeeding sampled either manually or using pumps were compared using one-way ANOVA tests. Kruskal-Wallis tests were applied when sample data were not normally distributed. All analyses were conducted with a significance level of *P*<0.05. Collected data were analysed using the statistical software package Statgraphics Centurion XVII version 17.0.16 (Statpoint Technologies Inc., Warrenton, VA, USA).

## Results

### Effect of the collection method (manual expression *vs*. pumping) on the milk microbial profiles

Initially, 60 women (30 with breast/nipple symptoms traditionally associated to “mammary candidiasis” and 30 without breastfeeding problems) were recruited in order to elucidate the influence of manual milk pumps on the milk microbial load and profile. The complete raw dataset is shown in S1 Table. The frequency and the values for the selective counts of the isolates belonging to the species *Staphylococcus epidermidis*, *Staphylococcus aureus*, *Streptococcus mitis/oralis*, and *Streptococcus salivarius* and to the genera *Rothia* and *Corynebacterium* were similar independently of the milk collection method in both groups (Table 1). In contrast, both the frequency and the mean concentration values for members of the Family *Enterobacteriaceae*, other Gram-negative bacteria, and yeasts (*C*. *albicans*) were significantly higher among samples obtained using their own milk pumps (Table 1). Enterobacteria were isolated from one (~1.7%) of the 60 women when the samples were obtained by manual expression but they were detected in 28 women (~47%) when they used pumps. Similarly, other Gram-negative bacteria (*Pseudomonas* and *Stenotrophomonas*) were absent in manually expressed milk but present in 26 (~47%) of the pump-collected samples. Finally, yeasts (*C*. *albicans*) were present in 5 (~8%) of the manual samples and in 25 (~42%) of the pump-collected samples. In addition, mean [95% CI] concentration of yeast in milk samples obtained by manual expression was about 2.16 [1.69; 2.63] CFU/mL while in those collected by women using a pump was about 3.84 [3.59; 4.10] CFU/mL (P<0.001; Kruskal-Wallis test), i.e. the use of pumps increased the yeast mean values by 1.68 (±0.76) log units.

**Table 1:** Microbiological analysis of milk samples from women with and without symptoms of presumptive “mammary candidiasis” after milk extraction by either manual expression or pumping.

**Women with painful breastfeeding(n = 30)**								
**Microorganism**	**Manual expression**			**Pump expression**			P-value*	P-value**
	**n (%)**^1^	**Mean [95% CI] or median (IQR)** **(log**_**10**_ **CFU/mL)**^2^	**min–max**^3^ **(log**_**10**_ **CFU/mL)**	**n (%)**	**Mean [95% CI] or median (IQR)** **(log**_**10**_ **CFU/mL)**	**min–max** **(log**_**10**_ **CFU/mL)**		
*Staphylococcus epidermidis*^4^	27 (90)	4.54 [4.34; 4.74]	3.46–6.18	26 (87)	4.48 [4.28; 4.68]	3.32–6.18	0.648	0.763
*Staphylococcus aureus*	3 (10)	3.63 [3.05; 4.21]	3.17–4.21	4 (13)	3.72 [3.22; 4.22]	3.32–4.54	0.202	0.839
*Streptococcus mitis/oralis*^5^	16 (53)	4.32 [4.07: 4.57]	3.26–5.67	15 (50)	4.40 [4.14; 4.66]	3.17–5.70	0.500	0.749
*Streptococcus salivarius*	12 (40)	4.21 [3.95; 4.47]	3.21–5.32	11 (37)	4.38 [4.11; 4.65]	2.95–5.21	0.500	0.518
Genus *Rothia*	6 (20)	2.62 [2.14; 3.10]	2.35–4.28	5 (17)	2.88 [2.31; 3.45]	2.49–4.35	0.500	0.596
Genus *Corynebacterium*^6^	5 (17)	3.45 [2.84; 4.06]	2.32–4.38	4 (13)	3.48 [2.80; 4.16]	2.41–4.41	0.500	0.958
*Enterobacteriaceae*^7^	1 (3)	2.18	-	13 (43)	4.63 [2.17; 6.00]	2.17–6.00	<0.001	-
Other Gram-negative bacteria^8^	0 (0)	-		14 (46)	3.16 [2.66; 3.67]	2.66–3.67	<0.001	-
Yeasts	4 (13)	2.21 [1.55; 2.88]	1.88–2.35	12 (40)	4.32 [3.94; 4.70]	2.27–5.54	0.020	<0.001
*Candida albicans*	4 (13)	2.21 [1.55, 2.88]	1.88–2.35	12 (40)	4.32 [3.94; 4.70]	2.27–5.54	0.020	<0.001
*Candida parapsilosis*	0 (0)	-		0 (0)	-	-	-	
*Saccharomyces cerevisiae*	0 (0)	-		0 (0)	-	-	-	
**Women without painful breastfeeding(n = 30)**								
**Microorganism**	**Manual expression**			**Pump expression**			P-value*	P-value**
	**n (%)**	**Mean [95% CI] or median (IQR)** **(log**_**10**_ **CFU/mL)**	**min–max** **(log**_**10**_ **CFU/mL)**	**n (%)**	**Mean [95% CI] or median (IQR)** **(log**_**10**_ **CFU/mL)**	**min–max** **(log**_**10**_ **CFU/mL)**		
*Staphylococcus epidermidis*	25 (83)	2.30 (0.42)	1.88–3.00	25 (83)	2.38 (0.16)	2.00–3.18	0.635	0.150
*Staphylococcus aureus*	2 (7)	1.97 [1.54; 2.40]	1.88–2.06	2 (7)	1.88 [1.44; 2.31]	1.70–2.06	0.694	-
*Streptococcus mitis/oralis*	12 (40)	2.41 (0.26)	2.00–2.90	11 (37)	2.26 (0.18)	1.97–3.00	0.500	0.185
*Streptococcus salivarius*	10 (33)	2.44 (0.13)	2.35–2.88	10 (33)	2.38 (0.08)	2.17–2.93	0.608	0.434
Genus *Rothia*	5 (17)	2.27 [2.05; 2.49]	1.95–2.72	4 (13)	2.17 [1.93; 2.41]	2.00–2.63	0.374	0.622
Genus *Corynebacterium*	3 (10)	1.96 (0.78)	1.88–2.67	3 (10)	2.72 (0.79)	1.97–2.72	0.665	0.127
*Enterobacteriaceae*	0 (0)	-	-	15 (50)	4.51 [4.19; 4.83]	2.10–6.18	<0.001	-
Other Gram-negative bacteria	0 (0)	-	-	12 (40)	3.00 [2.89; 3.11]	2.49–3.46	<0.001	-
Yeasts	4 (13)	2.11 [1.46; 2.77]	1.88–2.63	16 (53)	3.49 [3.16; 3.81]	1.95–5.72	0.001	0.012
*Candida albicans*	1 (13)	2.48	-	13 (43)	3.76 [3.41; 4.11]	2.27–5.72	<0.001	-
*Candida parapsilosis*	1 (13)	2.30	-	1 (3)	2.49	-	0.754	-
*Saccharomyces cerevisiae*	2 (7)	1.91 [1.46; 2.36]	1.95–2.24	2 (7)	2.20 [1.76; 2.65]	1.95–2.41	0.694	-

^1^n (%), number (percentage) of positive samples.

^2^Mean [95% CI]microbiological counts (when data were normally distributed)or median (IQR)microbiological counts (when data were not normally distributed); CI, confidence interval; IQR, interquartile range; CFU, colony-forming units.

^3^min, minimum value; max, maximum value.

^4^Other staphylococcal species that were isolated and identified included *S*. *capitis*, *S*. *haemolyticus*, *S*. *pseudintermedius*, *S*. *warneri*, *S*. *pasteuri*.

^5^Other streptococcalspecies that were isolated and identified included *St*. *agalactiae*, *St*. *anginosus*, *St*. *dysgalactiae*, *St*. *gallolyticus*, *St*. *mutans*, *St*. *pyogenes*.

^6^Corynebacterial species that were identified: *C*. *mucifaciens*, *C*. *aurimucosum*, *C*. *pseudodiphtheriticum*, *C*. *jeikenium*, *C*. *xerosis/amycolatum*.

^7^*Klebsiella pneumoniae*, *Klebsiellaoxytoca*, *Escherichia coli*, *Enterobacter* spp. and *Serratia* spp.

^8^*Pseudomonas* spp. and *Stenotrophomonas* spp.

*Fisher exact tests.

**One-way ANOVA (when data were normally distributed) or Kruskal-Wallis (when data were not normally distributed) tests.

**Fig 2:**
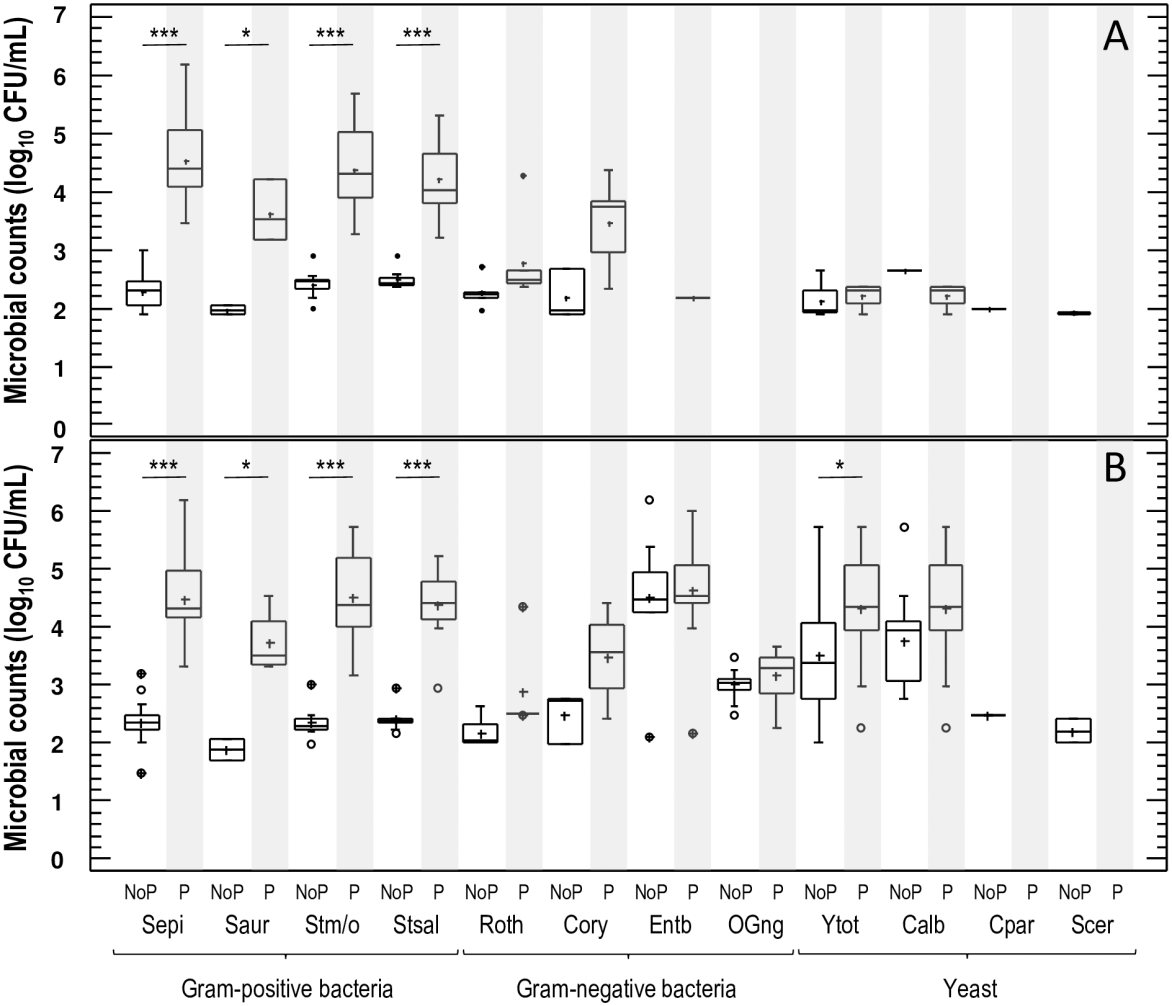
Microbial counts of milk samples obtained after milk extraction by manual expression (panel A) or pumping (panel B). Samples from women without painful breastfeeding are marked as "NoP" at the bottom of the figure while samples from women with painful breastfeeding are marked as "P" at the bottom of the figure and are shaded in grey. Abbreviations: Sepi, *Staphylococcus epidermidis*; Saur, *Staphylococcus aureus*; Stm/o, *Streptococcus mitis/oralis*; Stsal, *Streptococcus salivarius*; Roth, genus *Rothia*; Cory, genus *Corynebacterium*; Entb, *Enterobacteriaceae*; Ogneg, otherGram-negativebacteria; Ytot, yeasts (total); Calb, *Candida albicans*; Cpar, *Candida parapsilosis*; Scer, *Saccharomyces cerevisiae*. *, P<0.05; ***, P<0.001.

Substantial differences in the concentration (mean, minimum/maximum values) of some microbial genera and species were also detected between women with presumptive symptoms of “mammary candidiasis” and women with no painful breastfeeding. In fact, two-way ANOVA tests, with type of milk expression (manual/pump) and pain during breastfeeding (present/absent) as factors, indicated that having or not painful breastfeeding influenced significantly the value of microbial counts in the most abundant microorganisms found in milk samples, while the type of milk expression did not (Table 2). This trend was observed in *S*. *epidermidis*, *S*. *aureus*, *Streptococcus mitis/oralis*, *Streptococcus salivarius*, *Rothia*, and *Corynebacterium* counts. In contrast, yeasts and *C*. *albicans* counts varied strongly with the method of milk extraction (F = 24.22, P = 0.000 for yeasts, and F = 10.25, P<0.004 for *C*. *albicans*; two-way ANOVA) but they were not associated with painful breastfeeding (F = 1.76, P = 0.194 for yeast, and F = 0.02, P = 0.889 for *C*. *albicans*; two-way ANOVA) (Table 2). More specifically, the concentration of isolates belonging to the species *S*. *epidermidis*, *S*. *aureus*, *Streptococcus mitis/oralis*, and *Streptococcus salivarius* were higher (between 2.24 and 1.66 log units, depending on the species) among the samples provided by women with painful breastfeeding than in those obtained from healthy women (Table 1). These differences were observed both in samples extracted by manual expression or pumping (Fig 2).*Rothia* and *Corynebacterium* isolates were also detected more frequently, and at a higher concentration, in samples obtained from women with painful breastfeeding than in those provided from women without painful breastfeeding (Table 1, Fig 2), but the differences did not reach statistical significance (probably due to the low number of *Rothia*- and *Corynebacterium*-positive samples). In relation to yeasts, in contrast, differences in counts between samples from women with and without painful breastfeeding when the samples were extracted by pumping but not when they were obtained by manual expression (P = 0.032; one-way ANOVA) (Fig 2).

**Table 2:** Effects and interactions of the pain (presence/absence) during breastfeeding and the mode of extraction (manual/pump) of milk sampleson the microbial counts in milk samples as determined by two-way ANOVA tests.

Microorganism	Variables	*F*-value	*P*-value
*Staphylococcus epidermidis*	Pain	392.95	0.000
	Mode of extraction	0.03	0.870
	Pain × mode of extraction	0.50	0.479
*Staphylococcus aureus*	Pain	33.90	<0.001
	Mode of extraction	0.00	1.000
	Pain × mode of extraction	0.09	0.773
*Streptococcus mitis/oralis*	Pain	175.30	0.000
	Mode of extraction	0.00	1.000
	Pain × mode of extraction	0.28	0.599
*Streptococcus salivarius*	Pain	164.72	0.000
	Mode of extraction	0.08	0.783
	Pain × mode of extraction	0.81	0.373
Genus *Rothia*	Pain	4.83	0.043
	Mode of extraction	0.00	1.000
	Pain × mode of extraction	0.13	0.723
Genus *Corynebacterium*	Pain	9.46	<0.011
	Mode of extraction	0.21	0.655
	Pain × mode of extraction	0.14	0.713
Yeasts	Pain	1.76	0.194
	Mode of extraction	24.44	0.000
	Pain × mode of extraction	1.07	0.308
*Candida albicans*	Pain	0.02	0.889
	Mode of extraction	10.25	<0.004
	Pain × mode of extraction	0.93	0.345

It was noticeable that the 4 *C*. *albicans*-positive samples collected manually from women with painful breastfeeding were obtained from participating women whose infants were suffering from oral candidiasis (*muguet*). Within the same group of women, 8 *C*. *albicans*-positive samples were found when the samples were collected using pumps, however their infants were not suffering from oral candidiasis. Regarding women with no painful breastfeeding, yeasts (*C*. *albicans*, *Candida parapsilosis* and *Saccharomyces cerevisiae*) could be isolated from 4 participants (13%) by manual expression. Interestingly, they were the only participants that reported to suffer from type 1 diabetes. In the same group, use of pumps led to 16 *C*. *albicans*-positive samples from mothers without symptoms of diabetes or with children without oral candidiasis (Table 1).

Swab sampling of the internal surfaces and joints of the pumps (after regular cleaning) led to growth of enterobacteria, *Pseudomonas*, *Stenotrophomonas*, and/or *C*. *albicans* from all the devices that were positive for such microorganisms (data not shown). After observing the strong bias that pumping collection had on the microbial load and profile of the milk samples, it was established that the rest of the women that participated in this study (n = 529) had to collect the samples exclusively by manual expression using the procedure described by Arroyo et al. [16].

### Culture-dependent and–independent analysis of milk samples

The results obtained when the 529 additional milk samples were submitted to culture-dependent identification techniques (S2 Table) and genus-specific PCR analyses are shown in Table 3. *Staphylococcus* was the most frequently isolated bacterial genus; a total of 501 milk samples (95%) contained at least one isolate of this genus. The total staphylococcal counts found in the milk samples presented a median (IQR) value of 4.40 (0.92)log_10_ CFU/mL, ranging from 2.88 to 6.18log_10_ CFU/mL. *S*. *epidermidis* was the most common species isolated from the milk samples in this study (91%), while *S*. *aureus* was detected in 7% of the samples. *S*. *hominis* and *S*. *lugdunensis* were isolated in 7% and 5% of the samples, respectively. The median (minimum and maximum) counts for *S*. *epidermidis* were 4.34 (3.46–6.18)log_10_ CFU/mL, while the mean (minimum and maximum) counts for *S*. *aureus* were 3.72 (3.32–4.54) log_10_ CFU/mL(Table 3). *Rothia* was present in 23% of the samples, being *Rothia mucilaginosa* the species most commonly isolated of this genus, with a detection frequency of 18% (Table 3).

**Table 3:** Microbiological analysis (cultures and PCR assays) of milk samples from 529 women with presumptive symptoms of “mammary candidiasis” after milk extraction by manual expression.

Microorganism	Culture			Genus-specific PCR
	n (%)^1^	Median (IQR) or mean [95% CI] (log_10_ CFU/mL)^2^	min – max^3^ (log_10_ CFU/mL)	Positive n (%)^1^
(a) Genus *Staphylococcus*	501 (95)	4.40 (0.92)	2.88–6.18	507 (96)
*Staphylococcus epidermidis*	481 (91)	4.34 (1.06)	3.46–6.18	
*Staphylococcus aureus*	37 (7)	3.72 [3.56; 3.89]	3.32–4.54	
*Staphylococcus hominis*	37 (7)	4.10 (0.54)	3.47–5.30	
*Staphylococcus lugdunensis*	26 (5)	3.30 (0.53)	2.79–5.48	
Other staphylococcal species^4^	47 (9)	3.08 (1.22)	2.24–5.78	
(b) Genus *Streptococcus*	405 (77)	4.42 [4.35; 4.48]	2.95–6.04	455 (86)
*Streptococcus mitis/oralis*	243 (46)	4.40 [4.32; 4.48]	3.17–6.04	
*Streptococcus parasanguinis*	79 (15)	3.32 (0.20)	3.14–3.44	
*Streptococcus salivarius*	201 (38)	4.38 [4.29; 4.47]	2.95–5.70	
*Streptococcus vestibularis*	32 (6)	3.43 (0.36)	3.12–5.24	
Other streptococcal species^5^	63 (12)	3.36 [3.33; 3.40]	3.14–3.59	
(c) Other Gram-positive bacteria	187 (35)	2.95 (0.56)	1.94–5.18	
*Rothia mucilaginosa*	95 (18)	2.98 (0.54)	2.49–4.70	
*Corynebacterium tuberculostearicum*	26 (5)	2.24 (0.48)	1.97–4.15	
*Corynebacterium kroppenstedtii*	21 (4)	2.36 (0.36)	1.94–3.58	
Other corynebacterial species^6^	53 (10)	2.84 (0.24)	2.65–4.54	
Enterococci^7^	26 (5)	3.19 (0.35)	2.95–5.18	
(d) Genus *Candida*	11 (2)	2.18 [2,01; 2.35]	1.88–2.70	15 (2.5)
*Candida albicans*	11 (2)	2.18 [2.01; 2.35]	1.88–2.70	

^1^n (%), number (percentage) of positive samples.

^2^Median (IQR) microbiological counts (when data did not follow a normal distribution) and mean [95% CI] microbiological counts (when the data followed a normal distribution); IQR, interquartile range; CI, confidence interval; CFU, colony-forming units.

^3^min, minimum value; max, maximum value.

^4^Other staphylococci (<3% samples): *S*. *capitis*, *S*. *haemolyticus*, *S*. *pseudintermedius*, *S*. *warneri*, *S*. *pasteuri*.

^5^Other streptococci (<3% samples): *S*. *agalactiae*, *S*. *anginosus*, *S*. *dysgalactiae*, *S*. *gallolyticus*, *S*. *mutans*, *S*. *pyogenes*.

^6^Other corynebacteria: *C*. *mucifaciens*, *C*. *aurimucosum*, *C*. *pseudodiphtheriticum*, *C*. *jeikenium*, *C*. *xerosis/amycolatum*.

^7^*Enterococcus faecalis* (18 samples) and *E*. *faecium* (8 samples).

The total streptococcal counts in the samples had a mean value of 4.42 log_10_ CFU/mL and oscillated between 2.95 and 6.04 log_10_ CFU/mL. This genus was isolated from 77% of the samples and, therefore, constitutes the second most prevalent bacterial group in the analyzed milk samples. *S*. *mitis/oralis*, *S*. *salivarius*, *S*. *parasanguinis* and *S*. *vestibularis* were the most common species of this group, isolated in 46, 38, 15 and 6% of the samples, respectively (Table 3). A number of streptococcal isolates (n = 29) could not be identified at the species level.

Species belonging to the genus *Corynebacterium* were found in 14% of the specimens. *Corynebacterium tuberculostearicum* (5% of samples) and *Corynebacterium kroppenstedtii* (4% of samples) were the corynebacterial species most frequently isolated in this study. Finally, enterococci were isolated in 5% of samples, being *Enterococcus faecalis* the most common species of this group.

In relation to yeasts, *C*. *albicans* could be isolated from only 11 samples (2%) at a relatively low concentration (mean: 2.18 log_10_ CFU/mL; minimum-maximum: 1.88–2.70 log_10_ CFU/mL). As previously observed, the *C*. *albicans*-positive samples (n = 11) were obtained from participating women whose infants were suffering from oral candidiasis. Neither enterobacteria nor other Gram-negative bacteria could be isolated from the samples. Genus-specific PCR analysis showed that DNA from the genera *Staphylococcus*, *Streptococcus* and *Candida* could be detected in 507 (96%), 455 (86%) and 15 (2.5%) samples, respectively (Table 3). All the samples with isolates belonging to these genera gave a positive PCR reaction for the corresponding genus or genera. In addition, a few samples from which they could not be isolated also generated a positive PCR result, due to the higher sensitivity of the PCR technique.

In relation to the 393 women with painful nipples, direct contact of nipple and mammary areola on CNA, BC and SDC agar plates resulted in growth of cocci-shaped bacteria, mainly staphylococci and streptococci but yeasts were not isolated from any sample (data not shown).

### Microscopy analysis of the milk samples and the nipple biopsies

**Fig 3:**
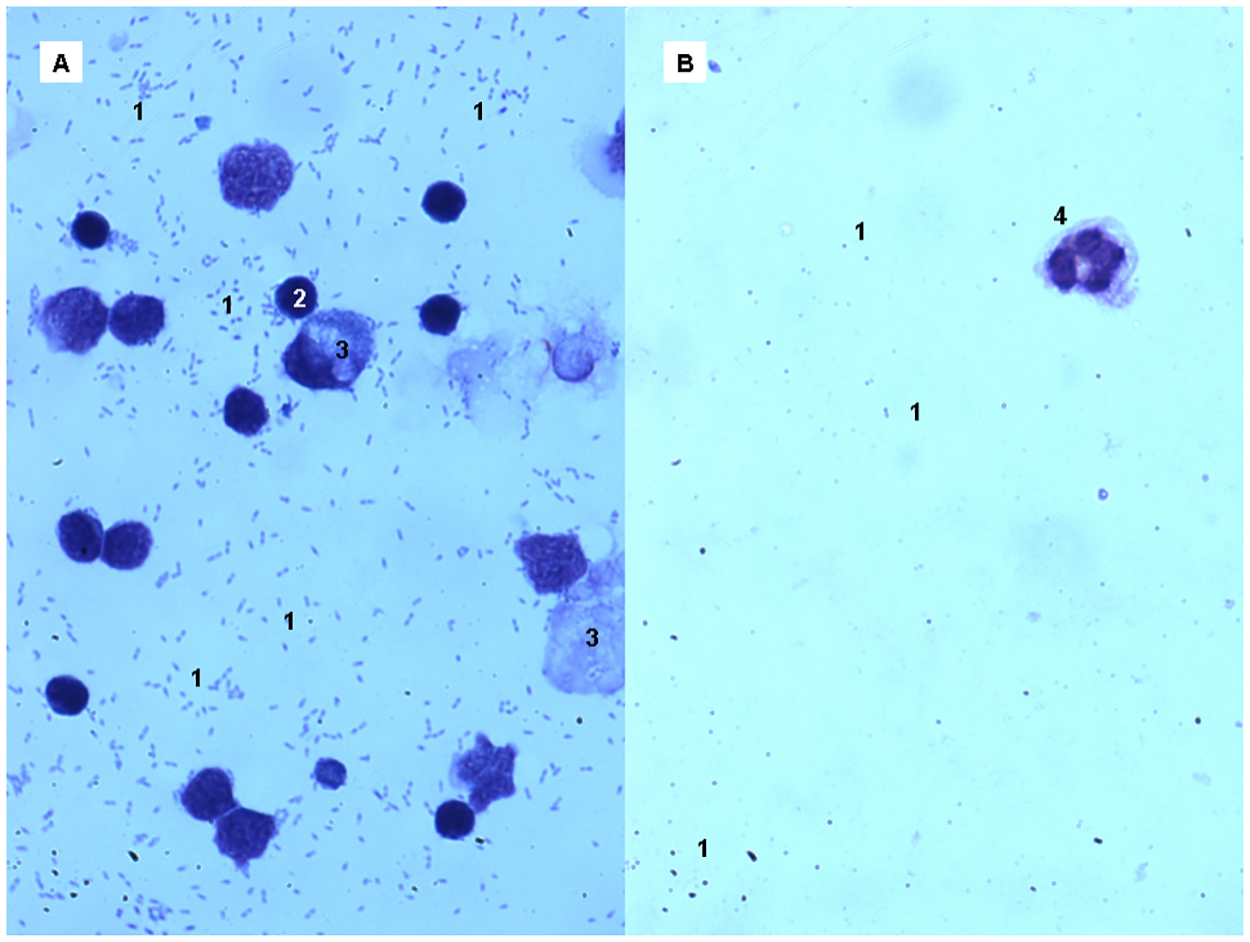
Optical microscopy observation of milk samples from a woman with presumptive signs of “mammary candidiasis” (A), and from another women without painful breastfeeding (B). 1, *Staphylococcus epidermidis* cells; 2, T cells; 3, epithelial cells; 4, polymorphonuclear neutrophil. Note the absence of yeasts’ cells.

The May-Grünwald-Giemsa stain was employed for the optical microscopy study of prokaryotic and eukaryotic cells in a subset of 260 fresh milk samples (~50%) provided by the participants with painful breastfeeding. The analysis of these samples revealed a high density of staphylococci and/orstreptococci, compatible with the results provided by culture-based methods. Neither *C*. *albicans* nor other yeasts could be observed in any tested sample. In addition, these samples showed a high density of somatic cells (T cells, epithelial cells) (Fig 3a). In contrast, control samples provided by healthy women (n = 24) were characterized by a low concentration of both bacterial and eukaryotic cells (Fig 3b).

**Fig 4:**
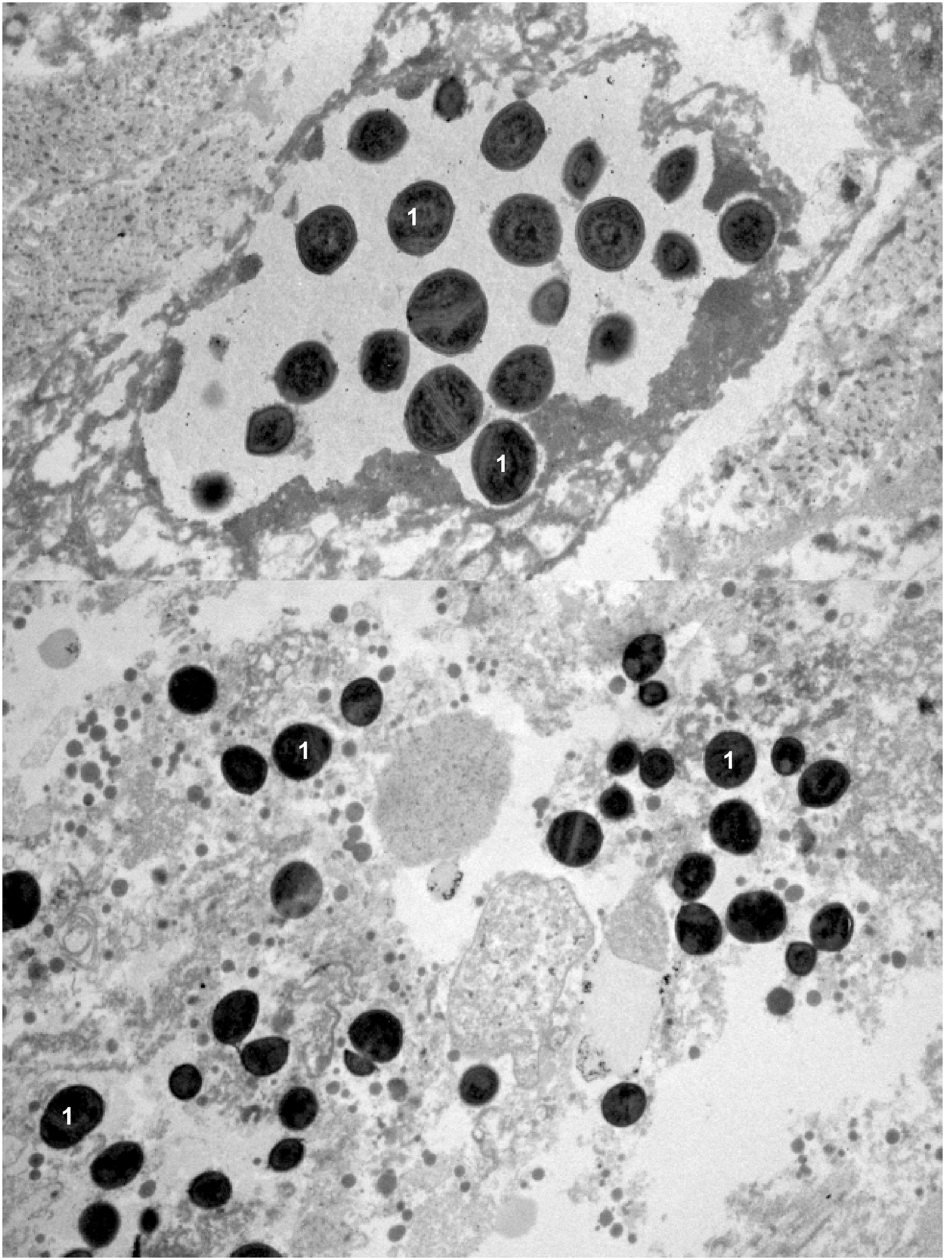
TEM images of two nipple biopsies from women with presumptive signs of “mammary candidiasis”. 1, *Staphylococcus epidermidis* cells. Note the absence of yeasts’ cells.

**Fig 5:**
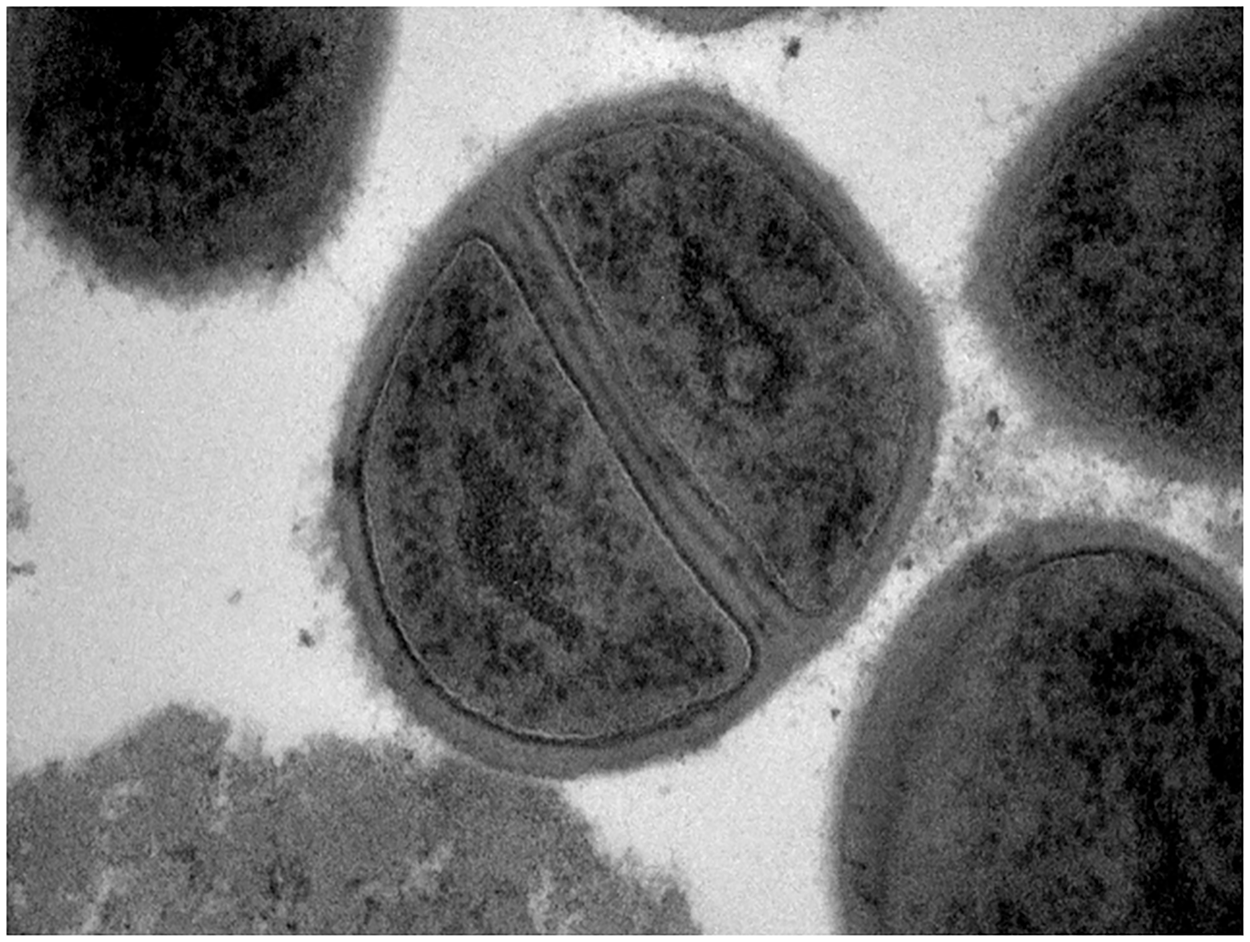
TEM image showing a closer view of rapidly dividing *Staphylococcus epidermidis* cells in a nipple biopsy from a woman with presumptive signs of “mammary candidiasis”.

TEM analysis of nipple biopsies provided by 25 women reporting sore, burning and/or painful nipples confirmed the presence of an inflammatory process, characterized by a high concentration of bacteria (mainly staphylococci and/orstreptococci) and the absence of yeasts (Figs 4 and 5).

## Discussion

In 2002, Foxman et al. [20] published a highly influential epidemiological study on the occurrence of lactational mastitis among almost 1,000 breastfeeding women, in which the authors highlighted how little is known about the etiology and pathogenesis of this common condition.

This situation has not changed substantially since then and it has allowed the persistence of non-scientific theories on the etiology of infectious conditions of the nipple and/or breast. Probably, the most widespread one supports that *Candida* species is responsible for the onset of sore or painful nipples and “shooting” breast pain in the absence of breast redness and flu-like symptoms, condition traditionally known as “mammary candidiasis or nipple trush” [4–9, 21]. Most surprisingly, such hypothesis is almost exclusively based on the visual assessment of the breast without the support of a proper microbiological analysis. In fact, a study on the diagnosis of thrush in the breastfeeding dyad found that 93% of the physicians surveyed in the study did not request laboratory testing [22].

The results of the present work clearly shows that no association could be established between painful breastfeeding (including shooting pain and sore nipples) and the presence of yeasts, both in milk or on the nipples. Such results were obtained by a great variety of microscopy techniques and culture-dependent and–independent identification methods applied to milk, nipple swabs and nipple biopsy samples provided by lactating women initially diagnosed as suffering from “mammary candidiasis” or “fungal infection” on the exclusive basis of visual assessment of the breast. Similarly, a rigorous microbiological study revealed that *C*. *albicans* was not present in the ductal tissues of breastfeeding mothers with the purported classic symptoms of ductal candidiasis [11]. Recently, a subset of 10 samples from the women recruited in this study (all of them reporting a painful breast/nipple) were the subject of a metagenomic analyses, showing that three classes were dominant: *Bacilli* (10–35% of the sequences), *Gammaproteobacteria* (17–32%), and *Alphaproteobacteria* (12–25%)[23]. The Genus *Staphylococcus* and the species *S*. *epidermidis*, mainly responsible for the mastitis as assessed by the milk cultures, accounted for 8–24% and 7–21%, respectively, of the reads obtained from these samples. Sequences from fungal, protozoan, archaeal and viral organisms were also detected and identified in the samples. Fungal reads belonging to the phyla *Basidiomycota* and *Ascomycota* were found in all the samples (0.23–1.15% of the total reads) while reads belonging to the genus *Malassezia* could be detected in some of them. In contrast, reads belonging to *C*. *albicans* or related species were not detected in any sample[23].

So far, the potential association between *C*. *albicans* and breastfeeding problems does not fulfill neither any of the revised Koch’s postulates nor any of the new criteria for establishing the etiology of infectious diseases [24, 25], including Hill’s epidemiological criteria for causal association (strength, consistency and specificity of association, temporality, biological gradient, plausibility, coherence, experimentation, analogy) [26]. Available scientific evidence does not support the *Candida*-breast/nipple association even when cultures searching for *Candida* spp. have been performed. A recent study supporting the “Candida hypothesis” [9] concluded that “*Candida* spp. is associated with burning nipple pain and breast pain” despite the fact that *Candida* spp. could be only isolated from 3% of the women (9 out of 346) with such symptoms.

In a different study, *C*. *albicans* was found more often in breastfeeding mothers who report pain compared with asymptomatic ones, but this microorganism could not be isolated from breast milk/nipple cultures of the majority (70%) of patients with pain [7]. It is interesting to address that the potential role of other microorganisms involved in breast pain, such as coagulase-negative staphylococci (CNS), streptococci or corynebacteria, was not taken into account in the design of any of the above-mentioned studies.

It must be noted that *Candida* spp. can be easily isolated in the laboratory when present in milk [11, 27]. Therefore, it is not surprising that many relevant studies have repeatedly pointed out the lack of scientific data supporting a potential relationship between fungal organisms and nipple/breast pain [11,12,28,29]. In addition, Dixon and Khan [30] warned that the treatment of lactating women with these symptoms with antifungal drugs (and without a proper etiological diagnosis) contributes to unnecessary overmedication and determines a low response to treatment and a high rate of recurrences and chronification of the condition.

In this study, *C*.*albicans* was isolated, at a low concentration, from samples provided by some mothers whose infants were suffering active oral candidiasis at sampling time. An association between staphylococcal/streptococcal mastitis and infant oral thrush may be established within a mother/infant dyad since such bacteria can induce *Candida* overgrowth. *C*. *albicans* and staphylococci/streptococci form a synergistic partnership where bacteria promote fungal growth and coaggregate leading to mixed-species biofilms [31–33]. After *C*. *albicans* overgrowth in the infant mouth, some of the yeast cells can be transferred to mother through breastfeeding, so that *C*. *albicans* could be isolated from breast milk and misdiagnosed as the cause of mastitis. As a consequence, mothers whose infants suffer oral candidiasis should not receive antifungal therapy, despite it is a regular practice [34].

Optical and TEM microscopy analyses of milk and/or nipple biopsy samples revealed the existence of an inflammatory process, a feature that is consubstantial with the term “mastitis”. Therefore, on the basis of the typical onset of the symptoms traditionally related to “mammary candidiasis”, and in order to distinguish it from acute mastitis (most often caused by *S*. *aureus*), the term “subacute mastitis” has been proposed to define such condition [35,36]. Such term is in agreement with the guidelines of the US National Library of Medicine (https://www.nlm.nih.gov) for the terminology of inflammatory and infectious diseases.

In relation to the etiology of “subacute mastitis”, the results provided by this and previous works [13–15, 36, 37] suggest that they may be caused by CNS, being *S*. *epidermidis* the species most often involved, and by streptococci (mostly mitis and salivarius groups). Occasionally, staphylococci related genera (e.g. *Rothia*), corynebacteria and enterococci may be also involved. In this study, the mean concentration of such microorganisms was significantly higher in samples provided by women suffering from subacute mastitis than in those provided by women with no painful breastfeeding. Further studies are required to establish casualty relationships.

CNS and mitis/salivariusstreptococci are normal inhabitants of the mammary ecosystem during lactation but different host, microbial and medical factors may support their overgrowth leading to subacute mastitis [36]. In contrast to *S*. *aureus*, most CNS and mitis/salivarius streptococci strains do not produce toxins responsible for acute local signs and systemic flu-like symptoms; therefore, local breast symptoms are generally milder and do not include breast redness. However, these bacteria are able to form thick biofilms inside the ducts, inflaming the mammary epithelium and forcing milk to pass through a progressively narrower lumen. An increased milk pressure on an inflamed epithelium results in a characteristic shooting, needle-like pain, often accompanied by a burning sensation and the false sensation of insufficient milk supply (hypogalactia). Eventually, bacterial biofilms may fill up some ducts, obstructing or blocking the milk flow and leading to a breast engorgement[36].

Among CNS, *S*. *epidermidis* is the species most commonly associated with lactational mastitis and its importance as agent for such condition is increasing worldwide [12, 13, 38]. In fact, inoculation of *S*. *epidermidis* strains isolated from human mastitis into the mammary glands of lactating mice leads to clinical and histological signs of mastitis [38]. This species, a commensal inhabitant of the healthy human skin and mucosal surfaces, is also a common nosocomial pathogen living at the edge between commensalism and pathogenicity, and has developed interesting strategies to transform into a notorious pathogen [39, 40]. CNS have also become the most common mastitis causing agents in ruminants and other mammals in many countries [41–44].

Some authors have pointed out the need to correlate clinical exam findings with culture results and therapy outcomes [45]. Milk cultures would help to identify potential pathogens and to provide targeted antimicrobial treatment [46]. It may seem a simple and practical approach but it is not an easy issue, due to the absence of standard protocols for the collection of this biological fluid, the doubts that often arise for the interpretation of the results and the lack of tradition in human milk microbiological analysis. The collection of a representative sample for microbial analysis is of outmost importance in order to reach a correct diagnosis since there are many sampling-related factors that may affect the result [16, 47]. Specifically, the use of milk pumps (and other devices) to collect the samples is associated to a high concentration of some contaminant bacteria (particularly enterobacteria, *Pseudomonas* spp., *Stenotrophomonas* spp. and related Gram-negative bacteria), and yeasts, that arise from the rinsing water, manipulations and other sources, but are not related to the particular mastitis case [48, 49]. Globally, there is a lack of standardized protocols for milk sample collection, storage and analysis in the scarce studies that reported microbiological data of milk cultures, and many of them did not considered the potential role of CNS, streptococci, corynebacteria or *Rothia* spp. as possible mastitis agents [16, 46, 50].

Unfortunately, in the few instances in which human milk cultures are performed, the above-mentioned microorganisms are usually regarded as “commensal” or “saprophytic” bacteria, independently of their concentration. Therefore, if there is a high concentration (>3 log_10_ CFU/mL) of these microorganisms in milk but, for example, *S*. *aureus* cannot be detected, the case is often wrongly reported as “non-infectious” mastitis. This makes that subacute mastitis have gone largely underrated, despite being the most frequent cause of mastitis, painful breastfeeding and precocious and undesired weaning.

In this work, a very rigorous clean-catch sampling method that removes cultivable *Candida* present in nipple or areola tissues, similar to the one used by Hale *et al*. [11], was used (Fig 2). Additionally, TEM analyses revealed that *Candida* spp. could not be observed in biopsies taken from the nipple’s region where women claimed to have a painful or burning feeling

In conclusion, the term “mammary candidiasis” should be avoided (at least in scientific peer reviewed journals) in relation to painful breastfeeding or sore nipples, as far as strong evidences are not presented, and replaced by subacute mastitis. Furthermore, it must be highlighted that a proper microbiological analysis is the only method that allows an etiological diagnosis of breast infection.

## Supporting information

S1 Table Microbiological analysis of milk samples from women with and without symptoms of presumptive “mammary candidiasis” after milk extraction by either manual expression or pumping. Counts (Log_10_ cfu/ml) of the main microbial species identified in the milk samples. See at the bottom of the table for abbreviations. (XLSX)
(XLSX)

S2 Table Counts (Log_10_ cfu/ml) of the microbial species identified in the milk samples (n = 529) analysed in this study. See at the bottom of the table for abbreviations. (XLSX)
(XLSX)
